# A synthetic lethal screen identifies HDAC4 as a potential target in MELK overexpressing cancers

**DOI:** 10.1093/g3journal/jkab335

**Published:** 2021-09-22

**Authors:** Lin Zhou, Siqi Zheng, Fernando R Rosas Bringas, Bjorn Bakker, Judith E Simon, Petra L Bakker, Hinke G Kazemier, Michael Schubert, Maurits Roorda, Marcel A T M van Vugt, Michael Chang, Floris Foijer

**Affiliations:** 1 European Research Institute for the Biology of Ageing, University of Groningen, University Medical Center Groningen, Groningen 9713 AV, The Netherlands; 2 Department of Medical Oncology, University of Groningen, University Medical Center Groningen, Groningen 9713 AV, The Netherlands

**Keywords:** MELK, HDAC4, genome-wide screen, synthetic lethality, cancer

## Abstract

Maternal embryonic leucine zipper kinase (MELK) is frequently overexpressed in cancer, but the role of MELK in cancer is still poorly understood. MELK was shown to have roles in many cancer-associated processes including tumor growth, chemotherapy resistance, and tumor recurrence. To determine whether the frequent overexpression of MELK can be exploited in therapy, we performed a high-throughput screen using a library of *Saccharomyces cerevisiae* mutants to identify genes whose functions become essential when MELK is overexpressed. We identified two such genes: *LAG2* and *HDA3*. *LAG2* encodes an inhibitor of the Skp, Cullin, F-box containing (SCF) ubiquitin-ligase complex, while *HDA3* encodes a subunit of the *HDA1* histone deacetylase complex. We find that one of these synthetic lethal interactions is conserved in mammalian cells, as inhibition of a human homolog of *HDA3* (Histone Deacetylase 4, HDAC4) is synthetically toxic in MELK overexpression cells. Altogether, our work identified a novel potential drug target for tumors that overexpress MELK.

## Introduction

Maternal embryonic leucine zipper kinase (MELK), a serine/threonine kinase, plays a dominant role in cell cycle regulation, proliferation, and apoptosis (Badouel *et al.* 2006; Jung *et al.* 2008). MELK is overexpressed in multiple human cancers, such as colorectal cancer (Ding *et al.* 2020), melanoma (Janostiak *et al.* 2017), and basal-like breast cancer cells (Wang *et al.* 2014) and is a cell cycle-regulated gene regulated by E2F transcription factors (Verlinden *et al.* 2005). Previous work has shown that MELK is involved in cytokinesis in *Xenopus* embryos and that MELK inactivation leads to cell division defects in this model (Badouel *et al.* 2010; Le Page *et al.* 2011; Tassan 2011). Furthermore, RNA interference and inhibitor studies have shown that inhibiting MELK activity in cultured mammalian cells suppresses tumor cell growth (Jiang and Zhang 2013; Wang *et al.* 2014, 2016). In mouse models, overexpression of wild-type MELK protein leads to oncogenic transformation, which relies on its kinase activity. Conversely, *in vivo* inhibition of MELK activity through the inhibitor OTSSP167 in breast cancer xenograft models revealed that inhibition of MELK activity reduced the growth of basal-like cell breast cancer xenografts, but not of luminal cell breast cancer xenografts (Wang *et al.* 2014). Similarly, OTSSP167 significantly improved the survival of mice in which A20 lymphoma cells were transplanted, which suggests that blocking MELK activity *in vivo* can also inhibit lymphoma progression (Maes *et al.* 2019). Germline inactivation of MELK in mice does not yield an obvious phenotype during embryo development or in adult mice, indicating that MELK is dispensable for normal development (Wang *et al.* 2014). Because of these combined observations, MELK is considered a highly selective cancer target.

Although previous studies provided evidence that MELK plays an important role in tumorigenesis, the precise role of MELK in tumor development has been challenged by more recent studies (Wang *et al.* 2014; Huang *et al.* 2017; Lin *et al.* 2017; Settleman *et al.* 2018). In particular, a study by the Sheltzer lab showed that CRISPR-mediated inactivation of MELK does not affect the fitness of triple-negative breast cancer cells, suggesting that OTSSP167 impairs cell division via off-target effects in these cells (Lin *et al.* 2017). Another study showed no difference between the *in vivo* growth of xenograft MDA-MB-231 cells in which MELK was inactivated or not (Giuliano *et al.* 2018). Therefore, the role of MELK in cancer is still not fully understood.

However, as MELK is so frequently overexpressed in human cancer (Nakano *et al.* 2008; Kuner *et al.* 2013; Wang *et al.* 2014; Speers *et al.* 2016; Xia *et al.* 2016; Chen *et al.* 2020), finding synthetic lethal interactions with MELK overexpression would still provide a powerful means to treat cancers that overexpress MELK. We therefore performed a high-throughput screen in *Saccharomyces cerevisiae* to identify genes that are required for yeast cell viability upon MELK overexpression. We followed up these findings in mammalian cells for two candidate genes, *LAG2* (encoding an inhibitor of the SCF ubiquitin-ligase complex) and *HDA3* (encoding a subunit of the *HDA1* histone deacetylase complex) (Marmorstein 2001; Yang and Grégoire 2005), and found that inhibiting the latter renders mammalian cells sensitive to MELK overexpression. While MELK overexpression did not alter cell proliferation, concomitant inhibition of HDAC4 significantly attenuated the proliferation of MELK overexpressing mammalian cells. Altogether, our work shows that MELK overexpressing cells are sensitive to the inhibition of HDAC4, which may provide novel intervention strategies to target tumor cells that overexpress MELK.

## Materials and methods

### Plasmids

To construct a doxycycline-inducible expression system for *MELK* overexpression, full-length cDNA encoding MELK was inserted between BamHI and NotI sites of three types of retroviral plasmids including the pRetroX-Tight-puro (Clontech), pRetroX-Tight-GFP-puro (containing N-terminal GFP), and pRetroX-Tight-BlastR (Puromycin exchanged by Blasticidin). The doxycycline-inducible pTRIPZ lentiviral shRNA vector targeting MELK (Catalog#: RHS4696-200691582) was purchased from Horizon Discovery. To generate inducible shRNA vectors targeting CAND1, oligonucleotides were selected from the Sigma RNAi Consortium shRNA library, annealed, and directly ligated into a gel-purified EZ-tet-pLKO-BlastR (Frank *et al.* 2017) backbone digested with NheI and EcoRI. To generate a constitutive shRNA vector targeting *HDAC4*, oligonucleotides were selected from the Sigma RNAi Consortium shRNA library, annealed, and directly ligated into a gel-purified PLKO.1-puro (SHC001; Sigma-Aldrich) backbone digested with AgeI and EcoRI. All vectors were verified by Sanger sequencing. All primers are listed in Table 1.

**Table 1:** Primers used in this study

Primer name	Sequence
MELK-F	CGCGCGGATCCATGAAAGATTATGATGAACTTCTCA
MELK-R	CGCCGGCGGCCGCTTATACCTTGCAGCTAGATAGGATG
Y-MELK(T167A)-F	AGGATTACCATCTACAGGCATGCTGTGGGAGTCTG
Y-MELK(T167A)-R	CAGACTCCCACAGCATGCCTGTAGATGGTAATCCT
hMELK-qPCR-F	GCCTGCCATATCCTTACTGG
hMELK-qPCR-R	GCCTCAATCTCCGTTTTGAT
hMELK-seq-F	CAGAGGCAGATGTTTGGAGCATG
hMELK-seq-R	CATGCTCCAAACATCTGCCTCTG
hTubulin-qPCR-F	CTTCGTCTCCGCCATCAG
hTubulin-qPCR-R	CGTGTTCCAGGCAGTAGAGC
PLKO.1-HDAC4-F	CCGGCAAGAATTTGTCCTCAATAAACTCGAGTTTATTGAGGACAAATTCTTGTTTTTG
PLKO.1-HDAC4-R	AATTCAAAAACAAGAATTTGTCCTCAATAAACTCGAGTTTATTGAGGACAAATTCTTG
HDAC4-qPCR-F	GAGAGACTCACCCTTCCCG
HDAC4-qPCR-R	CCGGTCTGCACCAACCAAG
EZ-PLKO-CAND1-F1	CTAGCTCCATAATCCAGAGGTTGTAACTCGAGTTACAACCTCTGGATTATGGATTTTTG
EZ-PLKO-CAND1-R1	AATTCAAAAATCCATAATCCAGAGGTTGTAACTCGAGTTACAACCTCTGGATTATGGAG
CAND1-qPCR-F	GCTGATATGTTGAGCAGGCAA
CAND1-qPCR-R	ACTGGGGAAGTAGACAGGTCA

To express human MELK in yeast, cDNA encoding human MELK was amplified by PCR. The resulting PCR product was cloned into a BamHI- and NotI-digested pSH380 plasmid, a *GAL1* promoter-containing pRS315-derived CEN plasmid (Vega *et al.* 2007). The kinase-dead MELK^T167A^ mutant was constructed using a Q5 site-directed mutagenesis kit (New England Biolabs).

### Cell lines and culture medium

Retinal pigmented epithelium (RPE1) cells, human mammary epithelial MCF10A cells, human mammary breast BT-20 cells, and MDA-MB-231 cells were purchased from the American Type Culture Collection (ATCC, Wesel, Germany). RPE1 p53 knockout cells were described earlier (Zomerman *et al.* 2018). RPE1, BT-20, and MDA-MB-231 cells were cultured in Dulbecco's Modified Eagle Medium (DMEM; Gibco, Carlsbad, CA, USA) supplemented with 10% fetal bovine serum (Invitrogen, Carlsbad, CA, USA) and 50 U/µl Penicillin/Streptomycin solution. MCF10A cells were cultured in DMEM/F-12 (Gibco, Carlsbad, CA, USA) supplemented with 5% horse serum (Invitrogen, Carlsbad, CA, USA), 50 U/µl Penicillin/Streptomycin, 100 ng/ml cholera toxin (Sigma), 20 ng/ml epidermal growth factor (Peprotech, Rocky Hill, CT, USA), 10 µg/ml insulin (Sigma), and 500 ng/ml hydrocortisone (Sigma).

### Time-lapse imaging

Time-lapse imaging was performed on a DeltaVision microscope (Applied Precision Ltd./GE). A total of 50,000 cells expressing pLNCX2 H2B-GFP were pre-seeded in four-well imaging chambers (LabTech). Cells were imaged with a DAPI-FITC-Cy5 filter set and images were captured every 4 min with a 40× objective. Filter specifications were as follows: for FITC, excitation: 475/28 and emission: 525/48, and for SiR-Hoechst, excitation: 632/22 and emission: 679/34. Mitotic abnormalities from the overnight videos were manually analyzed using softWoRxExplorer (Applied Precision Ltd./GE).

### Metaphase spreads

For metaphase spreads, cells were cultured up to 70% confluency with 10 µg/ml colcemid (Roche) for 3.5 h. Then, cells were harvested and incubated in 75 mM KCl for 10 min at 37°C. Next, cells were fixed in MAA (methanol:acetic acid; 3:1) and dropped on glass slides, then stained with Vectashield-DAPI (Brunschwig Chemie bv.). Metaphase images were inspected on an Olympus microscope using a 60× lens using a DAPI filter (excitation: 390/18; emission: 435/48).

### IncuCyte growth curves

Cells were seeded at a density of 40,000 cells/well in 12-well plates. Cell growth was monitored every 2 h by the IncuCyte Zoom live-cell analysis system (Essen BioScience Ltd.). Cell density was quantified by IncuCyte Zoom 2018A software (Essen BioScience Ltd.).

### qRT-PCR analysis

Total RNA was isolated and purified from cultured cells with the RNeasy Mini kit (Qiagen). A 1.5 µg of total RNA was reverse transcribed to cDNA in a 20-µl mixture of random primers, 10× RT buffer, RNase inhibitor, and reverse transcriptase (New England Biolabs). cDNA was quantified using the iTaq Universal SYBR Green supermix (Bio-Rad) on a LightCycler^®^ 480 instrument (Roche). Primers are listed in Table 1.

### Western blot

Cells were harvested by trypsinization and lysed in elution buffer (150 mM NaCl, 0.1% NP-40, 5 mM EDTA, 50 mM HEPES, pH 7.5) containing complete protease inhibitor (Roche) for 30 min. Samples were centrifuged at 300*g* for 10 min at 4°C to remove insoluble debris. Twenty microgram of each sample was loaded on 10% polyacrylamide gels and proteins were transferred onto polyvinylidene difluoride membranes. After blocking in Odyssey blocking buffer (Li-cor Biosciences) at 4°C for 30 min, the membrane was incubated overnight at 4°C with a primary antibody in blocking buffer. Following incubation, the membrane was washed with 1× PBS containing 0.1% Tween 20 (Sigma) for three times and incubated in secondary antibody for 1 h at room temperature. The blots were scanned using the Odyssey imaging system (Li-cor Biosciences). The protein bands were quantified with Image studio lite software (Li-cor Biosciences). We used the following antibodies: anti-MELK antibody (ab108529; Abcam), anti-α-Tubulin (ab7291; Abcam), anti-Mouse IRDye 680RD (P/N: 926-68072; Li-cor Biosciences), and anti-Rabbit IRDye 800RD (P/N: 926-32211; Li-cor Biosciences).

### Colony-formation assay

To assess proliferation potential, cells were seeded at a density of 40,000 cells/well in 12-well plates. After 5 days, cells were fixed with 4% formaldehyde and stained with crystal violet. To quantify relative cell numbers, 1 ml of 10% acetic acid was added per well to elute crystal violet, and absorbance was measured at a wavelength of 590 nm using a synergy H1 plate reader.

### Yeast strains and methods

**Figure 1:**
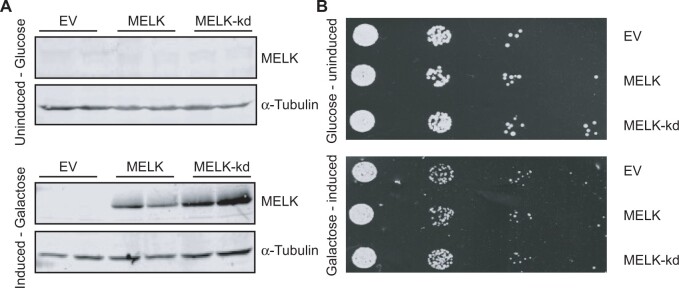
(A) Western blot on wild-type yeast lysates expressing an empty vector, MELK, and MELK-kd from a galactose-inducible promoter. MELK(-kd) is not expressed in the glucose medium (upper panel), MELK and MELK-kd are expressed in galactose medium (lower panel). Tubulin serves as a loading control. Two technical replicates from the same lysate were loaded per sample. (B) Serial tenfold dilutions of the indicated BY4741 yeast strains harboring either an empty vector, a plasmid expressing MELK, or a plasmid expressing MELK-kd were spotted onto synthetic medium-containing plates supplemented with either glucose or galactose.

Standard yeast media and growth conditions were used (Treco and Lundblad 1993; Sherman 2002). For the western blot analysis shown in Figure 1A, cells were taken from SD-leu (2% glucose-containing) plates and inoculated into either SD-leu or SGal-leu (2% galactose-containing) liquid media. The cultures were grown overnight, diluted into fresh media the next morning, and cultured until they reached mid-log phase before preparation of denatured extracts (see below). For the spot assays, cells were grown overnight in SD-leu media, then spotted onto either SD-leu or SGal-leu media following serial dilution. For the synthetic dosage lethality screen, the pSH380, pSH380-MELK, and pSH380-MELK-kd plasmids were transferred into the yeast knockout (YKO) library (Giaever *et al.* 2002) and a library of strains containing temperature-sensitive (ts) mutations of essential genes (Li *et al.* 2011) using a high-throughput method called selective ploidy ablation (SPA) (Reid *et al.* 2011), except that MELK or a kinase-dead variant of MELK (MELK-kd) was expressed from a galactose-inducible, instead of a copper-inducible promoter, allowing the elimination of the SPA pinning step onto copper-containing media. Replica-pinning steps were performed with a ROTOR HDA robot (Singer Instruments, Somerset, UK). The screen was performed in biological triplicate, and in each screen, every strain was present in quadruplicate. Colony growth data were processed using the ScreenMill software suite (Dittmar *et al.* 2010) to identify colonies with reduced growth upon expression of MELK or MELK-kd. Due to the unexpected inactivation of the web-based version of ScreenMill midway through our study, the third replicate screen was analyzed using the ScreenMill R package, which is publicly available through GitHub (https://github.com/EricEdwardBryant/screenmill; last accessed 09/27/21 ), resulting in a slight difference in the analysis of the replicate screens. For the first two replicate screens, strains whose growth on the experimental plate was 50% or less than the control plate were identified, followed by further selection using the SV Engine of ScreenMill. For the third replicate screen, putative hits were identified using a *P*-value cutoff of 0.1, which were then confirmed by visual comparison of plate images. The raw data of the putative hits from the replicate screens are contained in Supplementary File S1. A total of 255 putative hits were identified as having a growth defect upon expression of MELK at least once in the three replicate screens. Only 3 of the 255 hits were identified in more than one replicate screen. A subset of these hits was validated by the transformation of the three plasmids into strains from the YKO or ts libraries using the LiAc-based method (Schiestl and Gietz 1989), and their growth was tested by spot assays on synthetic medium lacking leucine containing either glucose or galactose. Only 2 (*lag2Δ and hda3Δ*) of 43 tested hits showed a robust growth defect upon MELK expression in the spot assays (Figure 4A; Supplementary File S3). The high false-positive rate was consistent with the poor overlap between the three replicate screens. To examine this further, we constructed a mini-array of strains consisting of almost all of the 255 putative hits and then re-screened this mini-array following the same SPA protocol we used for the genome-wide screen. Only 22 hits were reidentified, further illustrating the lack of reproducibility of this screen. One possible reason for this could be that MELK expression may have affected the screen, which involved the use of a plasmid-containing donor strain with 16 destabilizable centromeres and counterselectable chromosomes. Centromere destabilization in the SPA method relies in part on galactose-induced transcription through the centromeric regions, and since MELK/MELK-kd is also under the control of a galactose-inducible promoter, centromere de-stabilization and MELK/MELK-kd expression occurred simultaneously. Alternatively, the expression of human MELK may not impact yeast cell biology strongly enough to cause robust synthetic dosage lethal interactions. Regardless of the exact reason, the high false-positive rate indicates that a stricter threshold should have been employed to determine the putative hits.

### Preparation of denatured extracts of yeast

Ten milliliter of yeast culture grown to mid-logarithmic phase was centrifuged at 3000 rpm for 5 min at 4°C. The cell pellet was resuspended in 1 ml of 20% trichloroacetic acid (TCA; Sigma) and transferred to a FastPrep tube; cells were pelleted again and resuspended in 200 µl 20% TCA and glass beads for FastPrep were added. Yeast cells were lysed using FastPrep (MP Biomedicals) program: 6.0 m/sec 40 sec 1 cycle. Four hundred microliter of 5% TCA was added to the extract and the extract was collected by centrifugation at 1000 rpm for 1 min at 4°C. Then, the extract was centrifuged at 3000 rpm for 10 min, and the pellet was resuspended in 100 µl Laemmli buffer (150 mM Tris, pH 6.8, 6% SDS, 30% glycerol, bromophenol blue dye, 7.7% B-ME) and 50 µl 1 M Tris Base and boiled for 5 min. To remove debris, the tube was centrifuged at 300 rpm for 10 min at 4°C, and the supernatant was transferred to fresh protein LoBind tubes. Twenty microliter of the denatured extracts were loaded on polyacrylamide gels for subsequent analysis by western blot.

### Aneuploidy and MELK expression correlation

We downloaded both gene expression and copy number segments from The Cancer Genome Atlas (TCGA) breast cancer (BRCA) cohort via TCGAbiolinks (Colaprico *et al.* 2016) and showed the expression of MELK *vs* the aneuploidy score. We computed the expression level of MELK using the variance-stabilizing transformation of DESeq2 (Love *et al.* 2014), and the aneuploidy score as the average deviation of the mean DNA copy number measurements along the genome, excluding X and Y chromosomes.

### DepMap analysis

For a large set of cancer cell lines, gene-level essentiality scores [CRISPR (Clustered Regularly Interspaced Short Palindromic Repeats) or RNAi based], copy number, and mRNA expression data were obtained from DepMap release 21Q1 using the Broad Institute’s DepMap portal. Cell lines with fibroblast, teratoma, unknown, engineered, or of non-cancerous origin were excluded from the analysis. Pearson correlation coefficients were computed using R 4.0.0 to identify associations between MELK copy number or mRNA expression and gene essentiality scores. To correct for false positives, *P*-values were corrected using Benjamini-Hochberg correction.

## Results

### Identification of mutations sensitive to MELK overexpression

To identify genes that are required for cellular viability when MELK is overexpressed, we performed a synthetic dosage lethality screen in yeast. We chose to perform this screen in budding yeast, *S. cerevisiae*, because of the speed with which one can perform genome-wide screens and because many aspects of eukaryotic cell biology, including mitosis and chromosome segregation, are highly conserved in this model organism. For example, a similar strategy was successfully employed to identify synthetic dosage lethal interactions with CKS1B, which is frequently amplified in breast, lung, and liver cancers (Reid *et al.* 2016). We first tested whether expression of human MELK or MELK-kd is tolerated in yeast by introducing the human cDNAs under the control of the galactose-inducible *GAL1* promotor (Figure 1A). We found that both human proteins are well tolerated and that their expression does not noticeably affect cell growth in yeast in a spot assay (Figure 1B). We next introduced both plasmids into the YKO library, consisting of ∼4800 yeast strains in which each nonessential gene is deleted (Giaever *et al.* 2002), as well as a library of yeast strains containing ts alleles of essential genes (Li *et al.* 2011) and determined potential synthetic lethal interactions by quantifying the colony size of each mutant strain with or without MELK or MELK-kd expression. We performed this screen three times and found 255 potential synthetic lethal interactions, *i.e.*, mutant strains that grew slower when MELK was expressed, of which 214 grew slower only upon MELK, and not MELK-kd, expression (Supplementary File S2). When we analyzed the gene ontologies of these cumulative candidate genes, we found the enriched GO terms to be mainly involved in the biological processes such as “cell cycle,” “mitotic nuclear division,” and “cell division” (Supplementary File S2), which very much resemble findings of a previous yeast screen studying processes involved in chromosomal instability (CIN) (Stirling *et al.* 2011).

### Altered expression of MELK does not impair mitotic fidelity in mammalian cells

**Figure 2:**
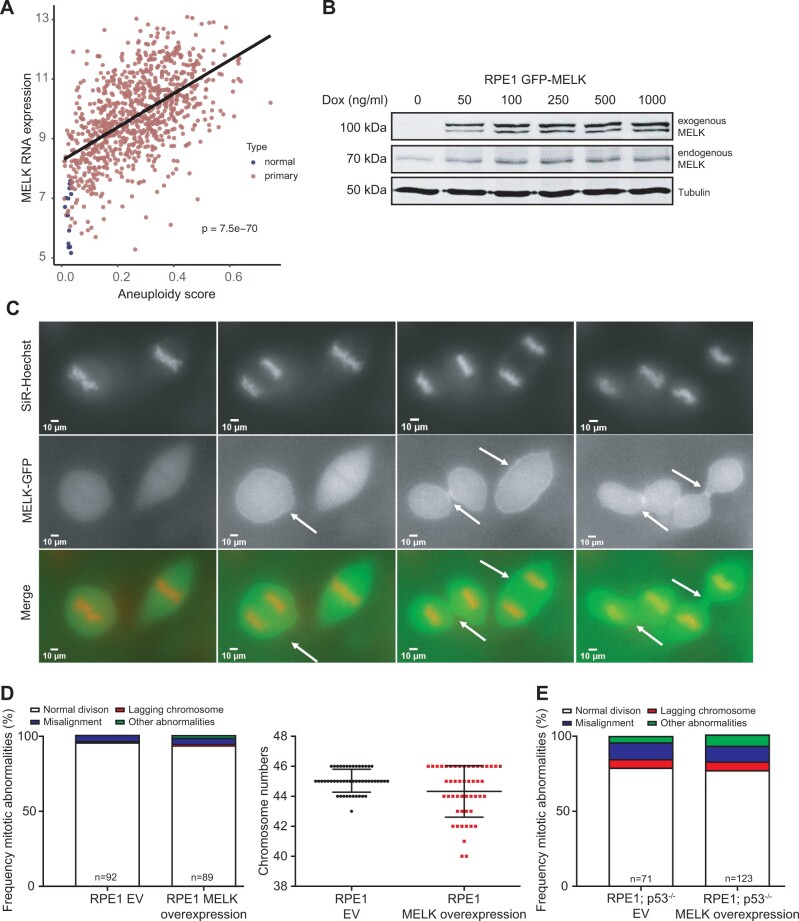
(A) Analysis of correlation between MELK expression and aneuploidy. MELK expression increases with higher aneuploidy levels in the TCGA breast cancer cohort. Aneuploidy is defined as the average DNA copy deviation from a euploid state. (B) Western blot of doxycycline-induced GFP-tagged MELK and endogenous MELK in RPE1 cells. Tubulin serves as a loading control. (C) Time-lapse imaging stills of mitotic RPE1 cells overexpressing GFP-tagged MELK showing that MELK localizes to the cleavage furrow during cytokinesis. Arrows indicate beginning (middle pane) and late cleavage furrow (right panel). (D) Frequency of mitotic abnormalities determined by time-lapse imaging (left panel) and quantification of karyotypes by metaphase spreads (right panel) in RPE1 cells with induced MELK overexpression. (E) Frequency of mitotic abnormalities determined by time-lapse imaging observed in MELK overexpressing RPE1 p53 knockout cells.

Since our synthetic lethal screen in yeast suggested a role of MELK in the control of mitosis and thus safeguarding genomic integrity (Supplementary File S2) in line with earlier observations in *Xenopus laevis* embryos (Tassan 2011), we next wanted to test whether MELK plays a role in maintaining genomic integrity in mammalian cells. We therefore determined whether MELK overexpression is correlated with aneuploidy in the TCGA database (Tomczak *et al.* 2015). Indeed, we found a strong positive correlation between MELK expression and aneuploidy (Figure 2A), suggesting that MELK is somehow involved in genomic instability. To better understand how increased expression of MELK is related to aneuploidy, we engineered a retina pigment epithelial (RPE1) cell line in which GFP-tagged MELK can be overexpressed under a doxycycline-inducible promotor. We first tested whether doxycycline addition indeed induced MELK-GFP expression and found that increasing doxycycline concentrations yielded higher MELK-GFP expression, which plateaued at 100 ng/ml of doxycycline. Quantification of the protein levels revealed that MELK-GFP was overexpressed 1.6-fold compared to endogenous MELK at this doxycycline concentration (Figure 2B).

**Figure 3:**
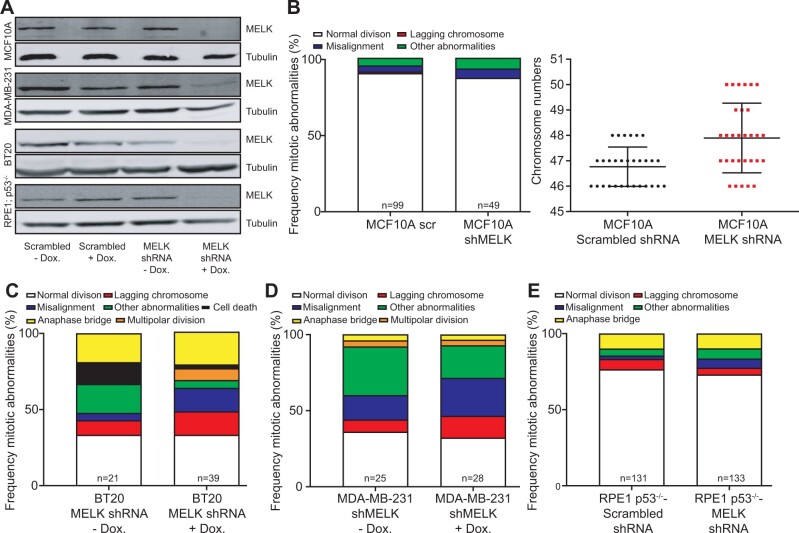
(A) Western blots for MELK protein showing doxycycline-induced MELK knockdown in MCF10A, BT-20, MDA-MB-231, and RPE1 p53^−/−^ cells compared to scrambled shRNA controls. Tubulin serves as a loading control. (B) Frequency of mitotic abnormalities determined by time-lapse imaging (left panel) and quantification of karyotypes by metaphase spreads (right panel) in MCF10A cells with MELK knockdown. (C–E) Frequency of mitotic abnormalities determined by time-lapse imaging observed in BT-20 cells (C), MDA-MB-231 cells (D), or RPE1 p53^−/−^ cells with MELK knockdown (E).

As MELK was previously shown to be involved in cytokinesis in *Xenopus* embryos (Tassan 2011) and mammalian cells (Chartrain *et al*. 2006), we first monitored MELK-GFP localization throughout the cell cycle by time-lapse imaging. We found, in agreement with other studies (Chartrain *et al.* 2006, 2013; Tipton *et al.* 2012), that MELK localizes to the mitotic cleavage furrow during cytokinesis in RPE1 cells (Figure 2C). We therefore next introduced the live-cell nuclear stain SiR-DNA (Spirochrome) into doxycycline-inducible MELK-GFP RPE1 cells to label the chromatin and used time-lapse imaging microscopy to investigate the effect of MELK-GFP overexpression in mitosis. We found that overexpression of MELK did not impair mitotic fidelity (Figure 2D), not even when we overexpressed MELK in p53^−^^/^^−^ RPE1 cells (Figure 2E) (Zomerman *et al.* 2018). Conversely, we also tested whether the inactivation of MELK would alter chromosome missegregation rates. For this purpose, we engineered doxycycline-inducible shRNA vectors targeting MELK, which we transduced into MCF10A, BT-20, and MDA-MB-231 cells as the latter two breast cancer cell lines overexpress MELK with MCF10A cells as a control. Western blots confirmed that MELK levels were reduced in all three cell lines (Figure 3A) compared to control cells expressing scrambled control shRNAs. We then introduced H2B-GFP by retroviral transduction in all shRNA-expressing cells and monitored cells by time-lapse imaging. This revealed that chromosome missegregation rates were not altered in any of the MELK knockdown cell lines (Figure 3, B–E), indicating that overexpression of MELK does not have a large effect on chromosome segregation. Altogether, our results suggest that neither overexpression of MELK nor its depletion significantly alters mitotic fidelity, even in a p53-deficient background.

### MELK overexpression sensitizes human cells to HDAC4 knockdown

**Figure 4:**
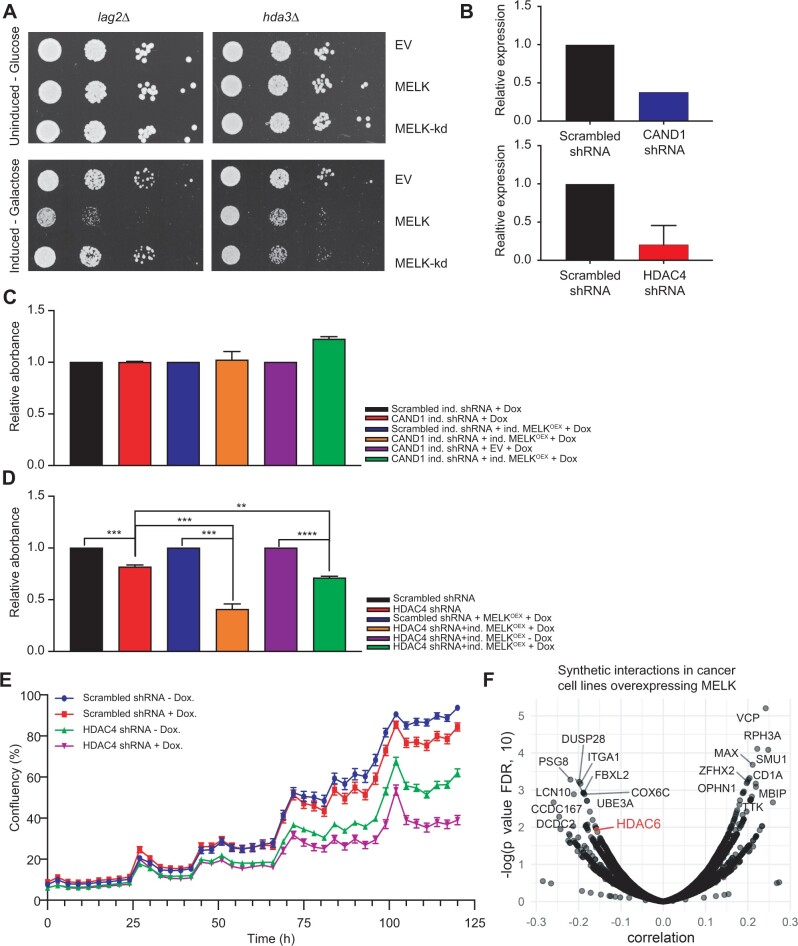
(A) Serial tenfold dilutions of indicated yeast strains harboring either an empty vector, a plasmid expressing MELK, or a plasmid expressing MELK-kd spotted onto yeast peptone (YP) medium plates containing either glucose or galactose. (B) RT-qPCR quantification of CAND1 (upper panel) and HDAC4 (lower panel) transcript levels before and after knockdown. Transcript levels were normalized to scrambled shRNA. (C) Quantification of colony formation potential of RPE1 cells with doxycycline-inducible (ind.) CAND1 knockdown compared to RPE1 cells expressing a control scrambled shRNA with or without doxycycline-inducible MELK overexpression. All statistics were calculated using GraphPad Prism software. Error bars indicate the mean ± standard deviation. (D) Quantification of colony formation potential of RPE1 cells with or without HDAC4 knockdown with or without MELK overexpression. All statistics were calculated using GraphPad Prism software. Error bars indicate the mean±standard deviation. *P*-values were analyzed using a two-tailed student’s *t*-test; ** refers to *P* ≤ 0.01; *** refers to *P* ≤ 0.001; **** refers to *P* ≤ 0.0001. (E) IncuCyte-determined proliferation curves showing growth of doxycycline-inducible MELK RPE1 cells expressing scrambled control shRNA with (red line) or without (blue line) doxycycline and doxycycline-inducible MELK RPE1 cells expressing an shRNA targeting HDAC4 with (purple line) or without (green line) doxycycline. Error bars indicate one standard deviation from the mean. (F) MELK mRNA expression correlates with HDAC6 RNAi knockdown-associated lethality in a large set of cancer cell lines (Pearson correlation coefficient = −0.1572, *P * = * *0.01186).

Since we did not find an effect of MELK overexpression or inhibition on chromosome segregation fidelity, we next decided to, instead of pursuing the role of MELK in chromosomal instability, pursue individual candidate genes that we had identified in the MELK synthetic dosage lethality yeast screen. We validated putative hits from our high-throughput screen by reintroducing the MELK expression plasmid, or the vector control, into a subset of the 255 putative hits, and spotting tenfold serial dilutions onto media containing glucose (non-inducing) or galactose (MELK-inducing). In total, only 2 of the 43 tested knockout strains showed a robust growth defect upon MELK expression; *lag2Δ* is sensitive to MELK, but not MELK-kd, expression, while *hda3Δ* is sensitive to both (Figure 4A). At present, we have no explanation for the high rate of false positives among our putative hits, especially given the enrichment of mitosis-related gene ontology (GO) terms. An extensive discussion about the high false-positive rate is given in the *Materials and methods* section.

Nevertheless, we decided to pursue Lag2 and Hda3 further as both have known human homologs CAND1 and HDAC4, respectively. *LAG2* is a longevity-assurance gene and its deletion significantly reduces the life span of yeast (Childress *et al.* 1996). Lag2 was found to be a negative regulator of the SCF complex (Liu *et al.* 2009), a role that appears to be conserved in mammalian cells through its homolog CAND1 (Zheng *et al.* 2002; Siergiejuk *et al.* 2009). Hda3 is essential for the activity of the yeast histone deacetylase Hda1 (Wu *et al.* 2001), similar to its mammalian homolog HDAC4, a Class II deacetylase that has a demonstrated role as a transcriptional repressor (Mielcarek *et al.* 2015).

To validate whether the genetic interactions observed in yeast are conserved in human cells, we engineered shRNAs targeting CAND1 and HDAC4, which we transduced into MELK overexpressing RPE1 cells. RT-qPCR confirmed reduced expression levels of CAND1 (∼60% knockdown) and HDAC4 (∼80% knockdown; Figure 4B). However, knocking down CAND1 did not alter cell proliferation in MELK overexpressing RPE1 cells nor control cells (Figure 4C).

When we next inhibited HDAC4 expression in control RPE1 cells, we found that this decreased colony formation by approximately 20% (Figure 4D, first two bars), but that this growth defect was much stronger in RPE1 cells that overexpress MELK (Figure 4D, middle bars, growth reduction of 60%). Importantly, a direct comparison between HDAC4 knockdown cells with and without MELK overexpression showed that MELK overexpression significantly delayed proliferation, both in a colony-formation assay (Figure 4D, last two bars) and in an IncuCyte proliferation assay (Figure 4E). We therefore conclude that the synthetic lethal interaction between loss of HDAC4/HDA3 expression and overexpression of MELK is conserved between yeast and human RPE1 cells.

Finally, we wanted to test whether our findings can be extrapolated to human cancer cell lines as well. We therefore downloaded expression data and copy number status of MELK from the Cancer Cell Line Encyclopedia (DepMap release 21Q1, also see *Materials and methods*) and tested which genes are essential in cells overexpressing MELK from RNAi screens. We found that, among other genes, inactivation of HDAC6, another type-II HDAC (Marampon *et al.* 2017), becomes more toxic when MELK is highly expressed, evidenced by a negative correlation between gene essentiality and MELK expression (Figure 4F). This observation confirms the findings of our screen and suggests that inhibition of type-II HDACs in general is particularly toxic in MELK overexpressing cells. Together, our results demonstrate that inhibition of HDAC4 activity impairs the proliferation of cells that overexpress MELK more than cells that do not overexpress MELK, thus revealing a potential targetable vulnerability of cancers that overexpress MELK.

## Discussion

MELK is frequently overexpressed in cancer, which makes it a promising target in cancer therapy. However, as MELK is an E2F target gene (Verlinden *et al.* 2005), its increased expression could well be a side effect of increased activity of E2F activity in cancer cells. This is well in line with the observation that MELK expression is positively correlated with the expression of several other proliferation markers such as MCM2, KI67, PCNA, CCNB1, and TOP2a (Giuliano *et al.* 2018). Therefore, it is unclear whether and how increased expression of MELK contributes to the fitness of cancer cells. While we do find that human cancers that overexpress MELK are significantly more aneuploid, we failed to find any direct effects of MELK overexpression on mitotic fidelity. Our findings therefore rule out a large effect of MELK overexpression on mitotic fidelity. Alternatively, our findings could indicate that additional, as of yet unidentified, predispositions together with MELK overexpression explain the strong correlation between aneuploidy and MELK overexpression.

We found that deletion of *LAG2* and *HDA3* renders yeast cells sensitive to the expression of MELK. However, the toxic interaction between loss of the *LAG2* homolog CAND1 and MELK overexpression was not conserved in mammalian cells. A possible explanation for this is that human cells may have additional pathways that can buffer the loss of CAND1. For instance, Lag2 (75 kDa) only regulates cullin in yeast, while its mammalian homolog CAND1 has a larger size (120 kDa) and also has functions beyond cullin regulation (Siergiejuk *et al.* 2009). An alternative explanation might come from the fact that our shRNA vector only reduced CAND1 by 60%, which might not have been sufficient to recapitulate the phenotype observed in yeast, in which the *LAG2* gene was completely inactivated. Finally, differences in the level of overexpression of MELK between yeast and mammalian cells could provide a possible explanation for the observed differences.

Depletion of the *HDA3* homolog HDAC4 histone deacetylase, on the other hand, did decrease the proliferation of MELK overexpressing cells. Importantly, this growth-inhibiting effect of HDAC4 inhibition was much stronger in MELK overexpressing cells compared to control cells. HDAC4 is a transcriptional repressor and its inhibition was found to reduce the growth of colon cancer cells through upregulation of p21 (Wu *et al.* 2001; Wilson *et al.* 2008; Mielcarek *et al.* 2015). Even though our work does not provide further mechanistic insight into the nature of the interaction between MELK and HDAC4, it is intriguing that MELK was identified as a *bona fide* interaction partner of HDAC4 in the BioGRID database (Boldt *et al.* 2016) and that HDAC6 inactivation becomes increasingly toxic with overexpression of MELK in cancer cell lines in the CCLE database (Figure 4F). Since both HDAC6 and HDAC4 have been shown to facilitate DNA damage repair in glioblastoma (Park and Kim 2020), the synthetic interaction between HDAC4 and HDAC6 with MELK could indicate a role for MELK in DNA damage as well. While our work provides functional evidence for a synthetic interaction between MELK overexpression and type-II HDACs, further experiments are needed to map the molecular mechanism underlying these interactions. Altogether, our work could provide a new angle of how to target MELK overexpressing cancers and might thus lead to novel intervention strategies in the future.

## Data availability

Strains, cell lines, and plasmids available upon request. Raw screening data is provided as Supplementary Dataset S1 and is available from figshare (https://doi.org/10.25387/g3.16607783). This data file contains all hits from the three replicates in the yeast screen generated by ScreenMill as explained in the readme sheet in Supplementary Dataset S1. The authors affirm that all data necessary for confirming the conclusions of the article are present within the article, figures, and tables.
